# Understanding and simulating the material behavior during multi-particle irradiations

**DOI:** 10.1038/srep30191

**Published:** 2016-07-28

**Authors:** Anamul H. Mir, M. Toulemonde, C. Jegou, S. Miro, Y. Serruys, S. Bouffard, S. Peuget

**Affiliations:** 1 CEA, DEN, DTCD, SECM, LMPA BP 17171, 30207 Bagnols-sur-Ceze Cedex, France; 2 CIMAP-GANIL (CEA-CNRS-ENSICAEN-Univ. Caen), BP 5133, 14070 Caen Cedex 5, France; 3 CEA, DEN, Service de Recherches de Métallurgie Physique, Laboratoire JANNUS, F-91191 Gif-sur-Yvette, France

## Abstract

A number of studies have suggested that the irradiation behavior and damage processes occurring during sequential and simultaneous particle irradiations can significantly differ. Currently, there is no definite answer as to why and when such differences are seen. Additionally, the conventional multi-particle irradiation facilities cannot correctly reproduce the complex irradiation scenarios experienced in a number of environments like space and nuclear reactors. Therefore, a better understanding of multi-particle irradiation problems and possible alternatives are needed. This study shows ionization induced thermal spike and defect recovery during sequential and simultaneous ion irradiation of amorphous silica. The simultaneous irradiation scenario is shown to be equivalent to multiple small sequential irradiation scenarios containing latent damage formation and recovery mechanisms. The results highlight the absence of any new damage mechanism and time-space correlation between various damage events during simultaneous irradiation of amorphous silica. This offers a new and convenient way to simulate and understand complex multi-particle irradiation problems.

There are a number of irradiation environments where materials are subjected to multiple particle irradiations over time. Typical examples are nuclear reactors (fission and fusion), nuclear waste disposal matrices (glasses and ceramics), spallation target vessels, space vehicles, extraterrestrial objects and a variety of radioisotope bearing minerals. To understand and model the irradiation behavior of the materials used in such environments and design innovative radiation resistant materials for future applications, it is important that correct irradiation experiments are employed to simulate these multiple particle irradiation environments. Therefore, double and triple beam irradiation experiments where materials are irradiated with two and three particles respectively, are becoming increasingly important and providing new insights into the radiation response of the pre-damage materials. Typical double beam (or any multiple-beam) irradiation experiment can be divided into sequential and simultaneous irradiation scenarios. Sequential double beam irradiation involves irradiating a material by two particles one after the other. The second irradiating particle therefore gives an idea about the response of the pre-damaged material. During simultaneous irradiation, the particles simultaneously irradiate the material, i.e. the particle beams are on at the same time (it does not imply that the actual damage events occur in the same space and time). The overall damage during simultaneous irradiation results due to the combined effects of the two particles and therefore it is not possible to isolate the nature and contribution of the individual particles towards the global damage formation.

Many sequential double ion beam irradiation studies of SiC^1^,^2^,^3^, MgO^3^, fluorapatite^4^,^5^,^6^ and glasses^7^,^8^ have shown that the pre-existing defects induced by the first particle can be annealed by the electronic ionization (LET) due to the second irradiating particle. On the other hand, molecular dynamics and sequential irradiation studies of SrTiO_3_^9^,^10^ and LiNbO_3_^11^ have shown that the pre-existing defects can enhance the ionization induced damage formation, commonly called as synergy. Therefore, depending on the nature of the material, defect recovery as well as synergistic effects can be observed. In addition to the sequential particle irradiations, a number of simultaneous particle irradiations have also been performed on various materials^1^,^3^,^7^,^12^. When comparing sequential and simultaneous irradiation results, no general conclusions about the interrelation between the two can be drawn from the studies available so far. Studies on SiC^1^,^2^,^3^, glasses^7^,^8^ and MgO^3^ have shown that the recovery effects are reduced during simultaneous particle irradiation in comparison to the sequential particle irradiation (double beam irradiation). Whereas studies on c-ZrO_2_^3^,^12^ and Gd_2_Ti_2_O_7_^3^ have shown no difference between the sequential and simultaneous particle irradiations.

A fundamental question that arises from these and many other studies^13^,^14^,^15^ is to know what makes a simultaneous irradiation scenario different from sequential or single beam irradiation scenarios and if there are any time-space correlation effects during simultaneous irradiation that modify the damage evolution process. If so, what are the governing conditions? The importance of this question lies far beyond mere differentiation of sequential and simultaneous irradiation scenarios. It is fundamental to addressing complex multi-particle irradiation scenarios for which even the irradiation facilities do not exist yet. For instance, exactly simulating a five particle simultaneous irradiation scenario consisting of neutrons, electrons, two different fission fragments of about 100 MeV energy and gamma rays (a typical irradiation scenario of cladding and nuclear fuel) is impossible with currently existing simultaneous irradiation facilities. Similarly, correctly simulating the effect of simultaneous beta and alpha decays is not possible as one is often restricted to using thin films which do not correctly represent the effects expected to occur in the bulk of the materials (e.g. nuclear waste glasses or multicomponent systems with highly mobile species). And at last, energy and flux limits at different multi-beam irradiation facilities make it difficult to simulate the high energy and low particle flux irradiation environments encountered in space and important to understand the radiation damage in spacecraft’s and extraterrestrial objects. Therefore, understanding what happens during simultaneous particle irradiations and exploring new ways of simulating complex irradiation problems is crucial to moving forward in this field. The present study provides answers to some of these questions and highlights the challenges in simulating complex irradiation scenarios involving multiple particles.

Detailed mono, sequential and simultaneous ion irradiations of amorphous silica with 2 MeV alpha particles and 14 MeV gold ions were performed. Amorphous silica was chosen as it constitutes a fundamental constituent of many glass systems used in different irradiation environments ranging from nuclear to space industry and a significant amount of research has already been done making available some vital experimental data and theoretical models. The alpha particles lose most of their energy through electronic energy loss causing electronic excitation and ionization. The gold ions on the other hand create significant ballistic damage resulting in atomic displacements in addition to the electronic energy loss. It is shown that the alpha particles result in damage formation in the pristine material and partial damage recovery in the gold pre-damaged material, whereas gold ions only result in the damage formation. Using thermal spike modelling, the recovery process is attributed to a low temperature non-melting thermal spike (600 °C) induced by the alpha particle ionization. Detailed sequential and simultaneous irradiation studies show that the compatibility between sequential and simultaneous irradiations depends on the pre-existing damage level as well as on the irradiation sequence of the ions. Therefore, care must be exercised when looking into the previous literature on this aspect. It is shown that the simultaneous irradiation is simply a sum of multiple small sequential irradiation scenarios. It contains a mixture of recovery and damage formation mechanisms exactly as they are present during the sequential irradiation scenarios. Therefore, there is no time-space correlation between the defects and no modification of the damage mechanisms or damage evolution occurs during simultaneous particle irradiation. The lack of any time-space correlation between various damage events, even at such high fluxes shows that the complex multi-particle irradiation environments can be easily simulated using classical single and double beam irradiation facilities.

## Results

### Raman spectroscopy: Qualitative differences in the spectra

#### Gold ion irradiation (Au)

**Figure 1:**
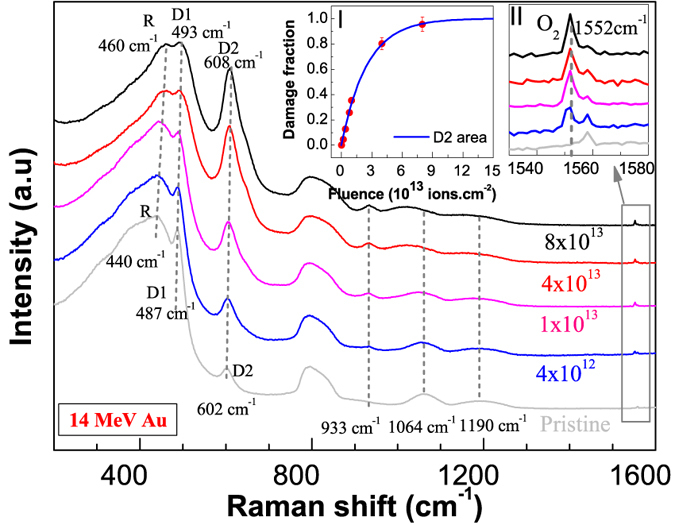
Non-polarized Raman spectra of a-silica irradiated with 14 MeV gold ions to various fluences. The fluences are written on the right extreme of the spectra in units of ions.cm^−2^. The spectra were normalized by the intensity of the R band (440 cm^−1^) and were vertically translated for better visualization in the figure above. The dotted lines highlight peak positions and shifts due to the irradiation. Inset-I shows the variation and saturation of D2 peak area with the irradiation fluence (normalized to 1). The line is a fit of single impact damage model (see section-2 in the SM for details about damage impact models). Inset-II shows the formation of molecular oxygen (1552 cm^−1^ peak). The less intense peak at 1558 cm^−1^ represents the atmospheric oxygen present between objective and the sample.

The energy loss and range of gold ions are shown in Table 1. The effect of 14 MeV Au ion irradiation on the structure of amorphous silica (a-silica) is shown in Fig. 1. The changes occurring in different peaks in the spectra are highlighted with dotted lines showing a modification of the network. Irradiation caused an increase in the frequency of the R band (bending of Si-O-Si bonds) from 440 cm^−1^ to 460 cm^−1^ (20 cm^−1^ increase). The population of four (D1 peak area) and three member rings (D2 peak area) also increased. The frequency of D1 and D2 peaks increased from 487 cm^−1^ to 493 cm^−1^ (6 cm^−1^ increase) and 602 cm^−1^ to 609 cm^−1^ (7 cm^−1^ increase) respectively. The peaks at 1064 cm^−1^ and 1190 cm^−1^ broadened and showed a downward shift in the frequency. New peaks emerged at 933 cm^−1^ and 1552 cm^−1^ whose intensity increased with the irradiation fluence. The 1552 cm^−1^ peak is attributed to molecular oxygen^16^ and is highlighted in inset-II (see section-1 in the Supplementary material (SM) for details about the origin and assignment of various peaks). Most of the structural changes saturated after irradiation with 4 × 10^13^ Au ions cm^−2^ (referred as saturation damage state) and only slight modifications were observed upon further increasing the fluence up to 8 × 10^13^ Au ions cm^−2^. The saturation behavior is shown in inset-I for the area of the D2 peak. This will be discussed in more detail in subsequent sections.

#### He irradiation (He)

**Figure 2:**
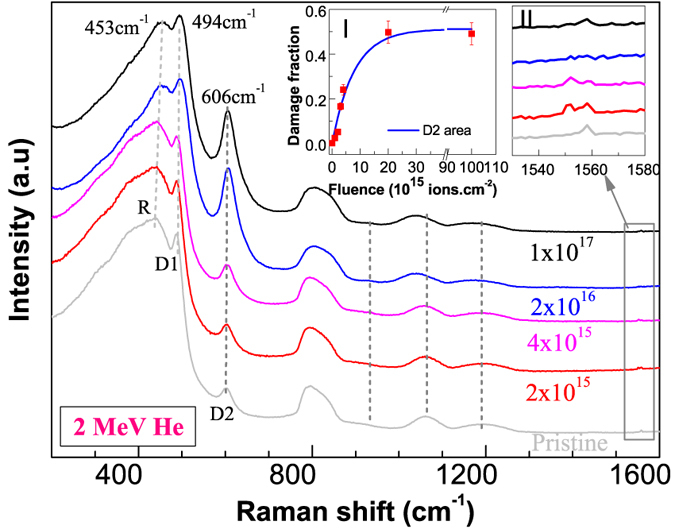
Non-polarized Raman spectra of a-silica irradiated with 2 MeV alpha particles to various fluences (ions.cm^−2^). Inset-I shows the variation and saturation of D2 peak area (the normalized area is shown relative to the gold ion irradiation). The line is a fit of single impact damage model. Inset-II shows the region of molecular oxygen. Unlike gold ion irradiation, no molecular oxygen was formed during alpha irradiation.

The effect of 2 MeV alpha irradiation on the structure of a-silica is shown in Fig. 2. The general trend is similar to what was observed during the gold ion irradiation, but, there are significant differences as well. The frequency of the R band increased from 440 cm^−1^ to 453 cm^−1^ only (13 cm^−1^ increase). The frequency of the D1 and D2 peaks increased from 487 cm^−1^ to 494 cm^−1^ (7 cm^−1^ increase) and 602 cm^−1^ to 606 cm^−1^ (4 cm^−1^ increase) respectively. Unlike gold ion irradiation, no new peak was observed at 933 cm^−1^ and there was only slight or no increase in the intensity of the molecular oxygen peak (1552 cm^−1^). The structural changes saturated after irradiation with 2 × 10^16^ He ions cm^−2^ (referred as saturation damage state). Increasing the fluence further by one order of magnitude did not have any further impact on the structural evolution. The saturation behavior of the D2 peak area is shown in inset-I. Inset-II shows the region of molecular oxygen.

From these structural changes one can infer that the gold ions and alpha particles resulted in different damage states. Alpha particles resulted in a preferential formation of four member rings (D1) over three member rings (D2), whereas gold ion irradiation showed an opposite behavior. Furthermore, gold ions resulted in a new defect type represented by a peak at 933 cm^−1^ and larger molecular oxygen formation. The Raman spectra of the saturation damage states observed during gold and alpha ion irradiations are compared in Supplementary Figure S1.

#### Sequential and simultaneous ion beam irradiations

**Figure 3:**
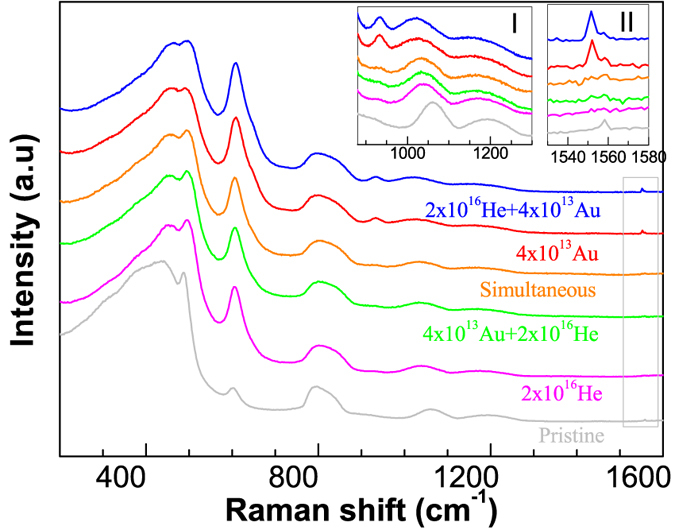
Non-polarized Raman spectra of a-silica corresponding to various irradiation scenarios (saturation damage states). The insets highlight the changes occurring in different regions of the spectra. Inset-I shows the region from 880 cm^−1^ to 1300 cm^−1^ containing 933 cm^−1^, 1064 cm^−1^ and 1190 cm^−1^ peaks. Inset-II shows the region of molecular oxygen formation (peak at 1552 cm^−1^).

In order to highlight the differences in the sequential irradiation scenarios and compare them with the mono ion beam irradiation scenarios, the Raman spectra of saturation damage states (4 × 10^13^ Au, 2 × 10^16^ He and their combinations of sequential irradiations) are shown in Fig. 3. Alpha irradiation of the gold pre-irradiated silica (4 ×10^13^ Au + 2 ×10^16^ He) caused simultaneous annihilation of the peaks at 933 cm^−1^ and 1552 cm^−1^ (compare red and green spectra in the main figure and insets). The D2 peak intensity and the position of R and D2 peaks decreased whereas an increase in the intensity of the D1 peak was observed. This resulted in a structural state resembling the saturation damage state induced by the alpha irradiation (compare pink and green spectra in the main figure and insets). Gold irradiation of the alpha pre-irradiated a-silica (2 ×10^16^ He + 4 ×10^13^ Au) on the other hand resulted in a new state resembling the Au ion irradiation induced saturation state (compare blue and red spectra. See also Supplementary Figure S2 for the comparison of two sequential irradiation scenarios only). Therefore, in general the second ion irradiation tends to remove any history related to the first ion irradiation and defines the final saturation damage state.

During simultaneous ion irradiation (orange spectrum), the Raman spectrum is intermediate between that of the alpha and gold irradiated samples. Nevertheless, the features specific to the gold ion irradiation (large D2 peak area, 933 cm^−1^ and 1552 cm^−1^ peaks) were significantly diminished and the Raman spectra resembled more closely to the alpha ion irradiation than to the gold ion irradiation (see also Figure S2 in the SM).

### Quantitative analysis of the Raman spectra

In order to track the damage evolution as a function of the irradiation fluence, it is important to follow the evolution of certain well defined features in the Raman spectra. The most distinct features that could be easily tracked as a function of the irradiation fluence are the R band position, D2 peak area and the area of the peak at 933 cm^−1^. The tendency of R and D1 bands to merge with an increase in the irradiation fluence introduced an ambiguity in their behavior at high fluence. For this reason, the area of the D2 peak, which is sufficiently isolated from rest of the peaks and sensitive to both types of irradiations was tracked as a function of the irradiation fluence. A straight line connecting two extreme points of the D2 peak was used for the background subtraction.

#### Mono and sequential ion beam irradiations

**Figure 4:**
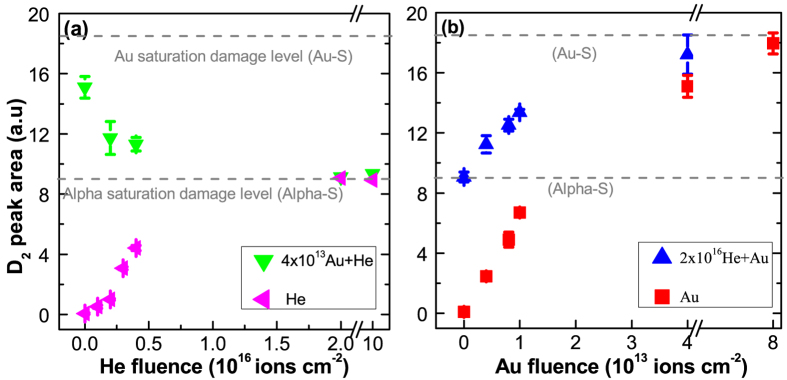
The evolution of D2 peak area during mono (He or Au) and sequential (He + Au and Au + He) ion irradiations. (**a**) Au + He irradiation sequence; Response of the pristine (pink-left triangle) and gold pre-irradiated (green-down triangle) a-silica to 2 MeV alpha irradiation; (**b**) He + Au irradiation sequence; response of the pristine (red-squares) and alpha pre-irradiated (blue-up triangle) a-silica to 14 MeV gold ion irradiation. Note that the saturation behaviour in (**b**) is less evident as compared to the inset-I in Fig. 1 due to the x-axis break between 4 × 10^13^ and 8 × 10^13^ in the above figure. The break was introduced to highlight the behaviour at low fluence.

The evolution of D2 peak area during alpha and gold ion irradiations of pristine a-silica is shown by pink and red data points in Fig. 4(a,b) respectively. Both, alpha and gold ion irradiations caused an increase of the D2 peak area. The area increased by 18 units during gold ion irradiation (taking the pristine level as zero). 85% of the damage level (15 units increase) was attained after irradiation with 4 × 10^13^ Au ions cm^−2^ whereas the 15% increase occurred much slowly from 4 × 10^13^ to 8 × 10^13^ Au ions cm^−2^. During He irradiation, the area of the peak increased by 9 units only. These are referred as the saturation damage levels and shown by dotted lines in subsequent figures.

The impact of alpha irradiation on the gold pre-irradiated glass (pre-irradiated by 4 × 10^13^ Au ions cm^−2^) is shown by green data points in Fig. 4(a). The 4 × 10^13^ Au ions cm^−2^ sample is almost fully damaged by the gold ions (85% of the saturation damage level). Therefore, its behavior during alpha irradiation reflects the response of the gold pre-damaged a-silica. The green data points clearly show that the alpha irradiation resulted in a partial repair of the pre-existing damage. The extent of the recovery depends on the alpha irradiation fluence. The final damage level is close to the saturation damage level observed during alpha irradiation of the pristine a-silica. The blue data points in Fig. 4(b) show the response of the alpha pre-irradiated a-silica to gold ion irradiation. Unlike alpha irradiation, no recovery effect was observed. The final state is close to the one observed during gold irradiation of the pristine a-silica. Therefore, Au irradiation of pristine and alpha pre-irradiated a-silica always induces damage formation.

**Figure 5:**
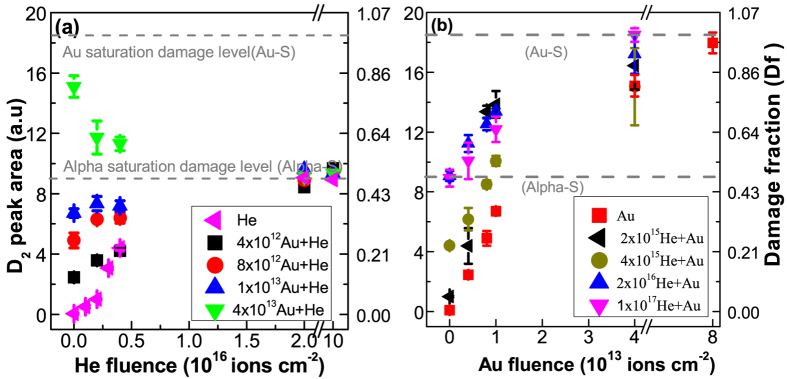
The evolution of D2 peak area during mono and sequential ion irradiations. (**a**) The evolution of D2 peak area during alpha irradiation (pink left triangle), 4 × 10^12^ Au + He (black squares), 8 × 10^12^ Au + He (red circles), 1 × 10^13^ Au + He (blue up-triangle) and 4 × 10^13^ Au + He (green down-triangle) sequential irradiations; (**b**) evolution of D2 peak area during Au ion irradiation (red squares), 2 × 10^15^ He + Au (black left-triangle), 4 × 10^15^ He + Au (dark yellow circles), 2 × 10^16^ He + Au (blue up-triangle) and 1 × 10^17^ He + Au (pink down-triangle) sequential irradiations.

Figure 5(a,b) show the irradiation response of a-silica pre-damaged to different damage levels with either gold or alpha particles respectively (the data of Fig. 4 is also shown). The left ordinate shows the D2 peak area and the right ordinate shows damage fraction (D2 peak area at a given fluence divided by the saturation D2 peak area. See section-2 in the SM for more details). For gold pre-irradiation fluences up to 1 × 10^13^ Au ions cm^−2^ (gold pre-damage level less than the alpha saturation damage level), sequential alpha irradiation showed overall damage buildup. For higher gold pre-irradiation fluence, sequential alpha irradiation showed damage recovery. This behavior results due to the fact that the response of the pristine and gold pre-irradiated a-silica to alpha irradiation is different (Fig. 4a). A competition between damage formation in the pristine part of the sample and damage recovery in the gold pre-damaged part of the sample defines the overall trend. The observed behavior therefore depends on the pristine fraction and the gold damage fraction; both of which change as a function of the gold pre-irradiation fluence. This will be discussed in more detail in the discussion part.

These results show that the role of the alpha particle depends on the pre-existing damage level. Alpha irradiation of the pristine a-silica leads to damage formation whereas the irradiation of highly pre-damaged one leads to partial damage repair.

#### Simultaneous (dual) beam irradiation and its comparison with other irradiation scenarios

**Figure 6:**
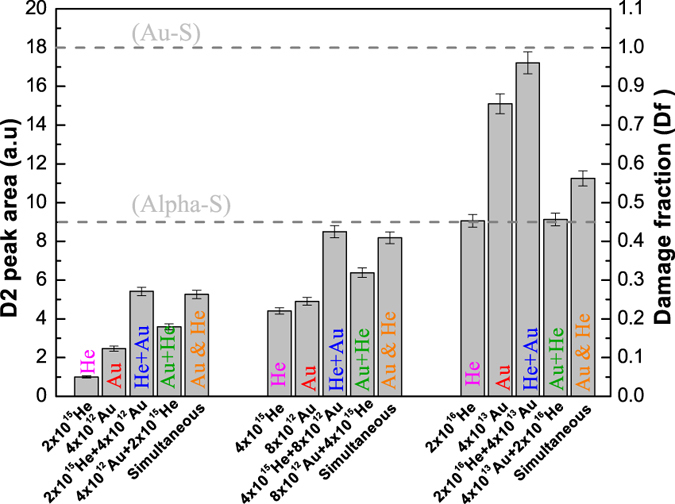
Comparison of D2 peak areas for various irradiation scenarios. The D2 peak area (left axis) and damage fraction (right axis) during different irradiation scenarios at low (left group), intermediate (middle group) and saturation (right group) fluence.

During this case, the samples were simultaneously irradiated with alpha and gold ions. Three simultaneous ion beam irradiation cases were studied (2 × 10^15^ He & 4 × 10^12^ Au, 4 × 10^15^ He & 8 × 10^12^ Au and 2 × 10^16^ He & 4 × 10^13^ Au). The D2 peak areas are shown in Fig. 6. The results of other relevant irradiation scenarios are also shown for comparison. For damage fraction (Df) <0.5, the damage level during dual ion beam irradiation is greater than the mono ion beam and Au + He irradiation scenarios and closer to the He + Au irradiation scenario. Therefore, the simultaneous and He + Au sequential irradiation scenarios are in good agreement with each other. For Df >0.5, the damage level during dual ion beam irradiation is intermediate between two mono ion beam irradiations and much closer to the Au + He irradiation scenario (see also Figure S3 in the SM which shows the intensity ratio of D2 and D1 for the saturation damage states). Therefore, Au + He and dual beam irradiation scenarios are in better agreement with each other. This shows that the compatibility between sequential and simultaneous irradiation scenarios depends on the pre-existing damage level and the irradiation sequence. This is an important point to bear in mind as many studies tend to compare sequential and simultaneous irradiation results without paying enough attention to the importance of the irradiation sequence and the pre-existing damage level.

#### Micro Hardness

**Figure 7:**
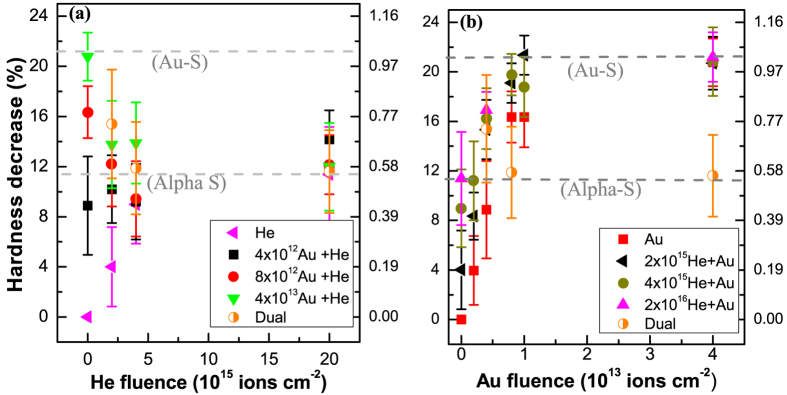
Variation of the micro-hardness under various irradiation scenarios. (**a**) Hardness variation during alpha irradiation (pink-left triangle) and Au + He sequential ion irradiation for different pre-existing gold damage levels (rest of the data); (**b**) hardness variation during Au ion irradiation (red-squares) and He + Au sequential ion irradiation for different pre-existing alpha damage levels (other data points). The right hand axes show the damage fraction.

Apart from the structural changes deduced from the Raman spectroscopy, the macroscopic effect was probed by measuring the changes in Vickers micro hardness of the irradiated samples. The evolution of the hardness of a-silica during various irradiation scenarios is shown in Fig. 7. Alpha and gold ion irradiations of pristine a-silica induced a hardness decrease of about 12% (Pink triangles in Fig. 7a) and 21% (red squares in Fig. 7b) respectively. Alpha irradiation of the gold pre-irradiated a-silica (Fig. 7a) showed recovery effect when the pre-damage level exceeded the alpha saturation damage level (case of 8 × 10^12^ and 4 × 10^13^ Au ions cm^−2^) and damage enhancement effect when the pre-damage level was lower than the alpha saturation level (case of 4 × 10^12^ Au ions cm^−2^). Gold irradiation of the alpha pre-irradiated a-silica did not show any recovery effect (Fig. 7b). These results essentially show the same trend as observed by the Raman spectroscopy and highlight that the response of a-silica to alpha irradiation depends on the pre-existing damage level (see Figure S4 in the SM for the comparison of various irradiation scenarios shown in the format of Fig. 6). It is however important to highlight that the hardness variation occurred faster than the variation of the D2 peak area. At a fluence of about 1 × 10^13^ Au ions cm^−2^, the D2 peak area had attained about 35% of the saturation value whereas the hardness has attained about 80% of the saturation value (see also Figure S5 in the SM showing the rate of variation of the hardness and D2 peak area).

## Discussion

### Mechanism of alpha induced damage repair

An interesting feature in the Raman spectra of Au + He irradiated a-silica is the annihilation of the molecular oxygen peak (1552 cm^−1^) and preferential formation of four member rings (and higher) over three member rings following alpha irradiation of the gold pre-irradiated a-silica. It is known that the molecular oxygen diffusion in a-silica and its recombination with E’ centers (a defect involving an oxygen vacancy) is activated at temperatures from 450 to 500 K^17^,^18^ (see also ref. 19 for defect annealing and defect inter-conversions in a-silica). The oxygen vacancy annihilation by O_2_ is known to be exothermic with an energy release of 4.5 to 5.5 eV and only diffusion limited without any reaction barrier^20^. Therefore, a first approach to address the alpha induced damage repair in a-silica is to look from the perspective of thermal spike formation upon alpha irradiation and annealing as a result of the alpha induced thermal spike.

**Figure 8:**
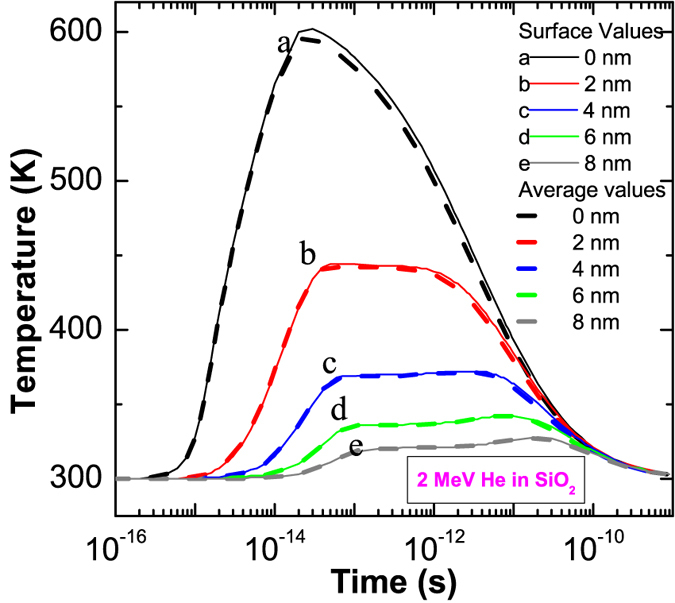
Thermal spike calculation for 2 MeV alpha particle in amorphous silica. The distance in nanometres shows the radial distance from the centre of the ion impact. The average temperature (dashed lines) was calculated by performing the thermal spike calculations using average total stopping power in the 2 μm depth and surface temperature (solid lines) calculation was performed using total stopping power on the sample surface (see Figure S6 in the SM for the calculations of 14 MeV Au ion).

Thermal spike calculations for 2 MeV alpha particles are shown in Fig. 8 (see Figure S6 in the SM for the calculation of 14 MeV Au ions). The calculations were performed using already validated parameters for a-silica^21^. The calculation is shown for an electronic stopping power value near the surface (surface values-solid lines) and for the average electronic stopping power in a 2 μm depth; which is the Raman depth resolution (average values-dashed lines). As the electronic stopping power of 2 MeV alpha particles does not vary significantly in the 2 μm depth, therefore both give same temperature profiles. The calculation shows that temperatures up to 600 K can be reached in the vicinity of the alpha ion impact. This temperature is strikingly close to what is usually needed for the diffusion and annihilation of oxygen vacancies and E’ centers in a-silica. The important thing to know is if such a diffusion process can occur at the typical time scale of a thermal spike (~10^−12^ seconds). The diffusion of oxygen within the glass structure depends on its chemical form. From the data available in the literature, the diffusion coefficient of molecular oxygen in amorphous silica^22^ is about 10^−3^ A^2^.ps^−1^ and that of the atomic oxygen^23^ is about 10 A^2^.ps^−1^. It suggests that the diffusion and recombination of the molecular oxygen during the time period of a typical thermal spike is much less feasible than that of the atomic oxygen. Therefore, the most likely mechanism is alpha induced ionization of the molecular oxygen into atomic species and subsequent diffusion and recombination of the atomic oxygen with the defects. The ionization of molecular oxygen to atomic oxygen in amorphous silica is known to occur under laser excitation for energies greater than 5.1 eV (UV lasers)^24^. The high electronic energy loss of the alpha particles in comparison to the photons can induce more efficient ionization than the photoionization and therefore represents a viable process (see section-1 in the SM for additional details).

### Damage evolution during sequential ion irradiations

The changes observed during sequential ion irradiations provides a cumulative information about three contributions: a) damage formation due to alpha irradiation of the pristine material; b) damage formation due to gold irradiation of the pristine material and c) damage formation due to gold irradiation of the alpha pre-irradiated material during He + Au irradiation sequence or d) partial damage recovery due to alpha irradiation of the gold pre-irradiated material during Au + He irradiation sequence. Unlike sequential ion irradiations where only three contributions can be present (viz: a, b and c or d), all four contributions from a-d are present during simultaneous ion beam irradiation. The cumulative damage level therefore depends on the damage fraction due to each ion and the degree of overlap between different damage zones.

**Figure 9:**
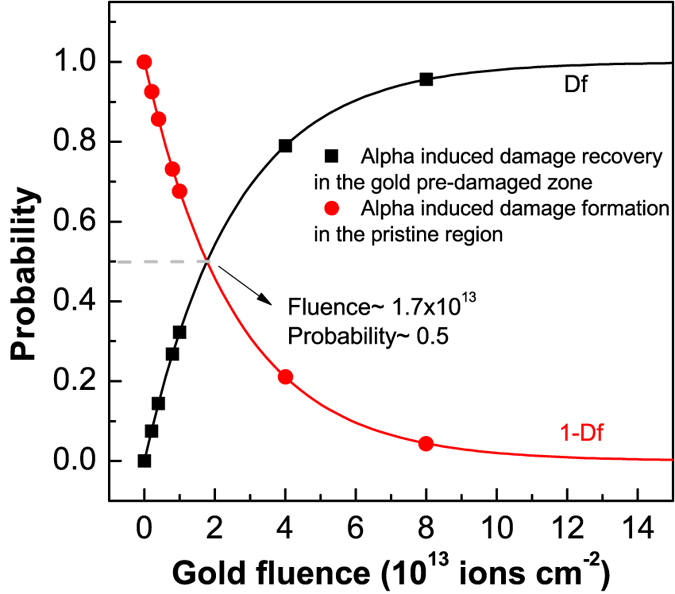
Probability to encounter pristine and gold pre-damaged zones. The probability of an alpha particle to encounter a gold pre-damaged zones is shown by black line (squares) and the probability to encounter a pristine zone is shown by red line (circles). Alpha irradiation of the gold pre-damaged zone causes partial damage recovery and that of the pristine zone causes damage formation.

The damage evolution (damage fraction-D_f_) during gold ion irradiation can be modelled using single impact damage model (see section-2 in the SM for more details). This is shown by the black line in Fig. 9 for the evolution of D2 peak area. D_f_ close to 1 means that the material is completely transformed by the gold ions whereas D_f_ close to 0 represents pristine material. During a sequential Au + He irradiation, the probability of alpha particles to hit a gold pre-damaged zone and induce damage recovery is proportional to D_f_, whereas the probability to hit a pristine zone and result in damage formation is proportional to 1-D_f_ (red line in Fig. 9). At any fluence one has to calculate the probability of encountering the pristine and pre-damaged regions and estimate whether the net damage formation in the pristine material is a dominant phenomenon or net damage recovery in the pre-damaged regions is dominant. This depends on the pre-damage fraction and the extent of the recovery caused by the alpha particles. The competition between damage formation in the pristine material and damage recovery in the gold pre-damaged material defines the macroscopic trend observed in the data in Figs 5 and 7.

Relative to the gold ion, if “r” and “d” represent recovery and damage levels (0 < r, d ≤ 1) respectively due to the alpha irradiation, then an equilibrium state can be obtained (no net damage formation) when;


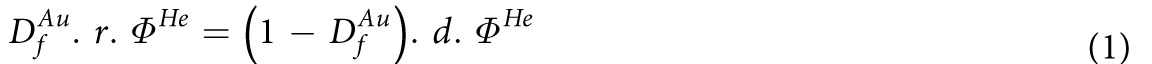


Therefore, for the equilibrium state;


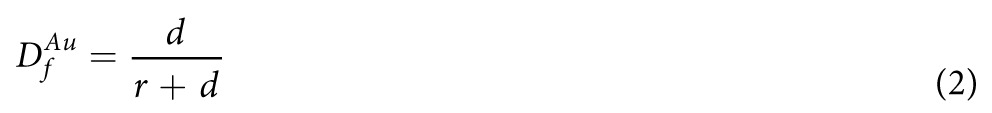


The trend will change from net damage formation to net damage recovery when 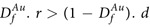. The dashed lines in Fig. 4 to 6 show alpha and gold saturation damage levels (the damage fraction on the right hand axes shows it on a scale of 0 to 1). Alpha irradiation of the pristine a-silica induced only half of the damage level in comparison to the Au ion irradiation, and alpha irradiation of the gold pre-irradiated a-silica caused a damage recovery by about 50% (D2 area decreases from 18 units to 9 units; i.e. d = r = 0.5). Therefore, the recovery effect becomes dominant and macroscopically visible when the probability for alpha particles to encounter gold pre-damaged a-silica exceeds 0.5. This occurs for gold pre-irradiation fluences greater than 1.7 × 10^13^ (shown in Fig. 9). For fluences lower than this, alpha irradiation causes a net damage buildup in the pristine a-silica and any recovery effect is masked. This is reflected in the data shown in Fig. 5(a). For gold pre-irradiation fluence of about 1.7 × 10^13^ a stationary state would be perceived despite the fact that the damage evolution is dynamic with damage recovery occurring in the gold pre-irradiated material and damage buildup in the pristine material. It is important to bear in mind that such description is only valid as long as the experimentally probed zone is greater than the damage zone of an individual ion. The impact of a single ion typically varies from angstrom scale to a few nanometers (typical size of the ion tracks).

For a given gold pre-damage level, the recovery effect continues until all the material attains the alpha induced saturation level. The difference between Au + He and He irradiation scenarios is maximum for the lowest fluence case (Fig. 6) because the gold damage fraction and the overlap probability are small (~0.1). The difference approaches zero at the highest fluence (damage fraction and overlap probability ~ 0.9) because of the higher degree of overlap and recovery.

The hardness behavior seen in Fig. 7 is also due to the competition between damage buildup in the pristine material and partial recovery in the gold pre-irradiated material. More details are given in Figure S7 and the associated description in the Supplementary material.

### Damage evolution during simultaneous ion beam irradiation

During simultaneous ion beam irradiation the cumulative damage results due to four contributions as mentioned earlier. At low fluence values, the overlap probability is low, therefore, no significant recovery occurs and the dual beam irradiation scenario is close to the He + Au irradiation scenario. At high fluences, the overlap probability is large and efficient recovery due to alpha particles leads to a close agreement between Au + He and dual beam scenario (Fig. 6).

If it is assumed that the particle fluxes are low enough such that no two ion impacts occur at the same place in a time period corresponding to the life time of the defects from the previous event, then any perturbation resulting due to time overlap of the defects from the two damage events can be ruled out. Under this assumption, simultaneous irradiation scenario can be assumed to be a result of the overlap of multiple random sequential irradiation scenarios. In other words, it means that if a material is subjected to simultaneous irradiation with four particles A, B, C and D up to fluences *ϕ*_*A*_, *ϕ*_*B*_, *ϕ*_*C*_, and *ϕ*_*D*_ respectively, then such an irradiation scenario is equivalent to (Δ*ϕ*_*A*_ + Δ*ϕ*_*B*_ + Δ*ϕ*_*C*_ + Δ*ϕ*_*D*_)_n_, where the irradiation sequence is repeated “n” times such that the fluence of each particle is incremented “n” times to attain the same fluence level as the one during the simultaneous ion irradiation (i.e. n*Δ*ϕ*_*A*_ = *ϕ*_*A*_). It was shown earlier that the last irradiating ion of the sequence usually defines the final damage state. Therefore, the **S**imultaneous **I**on beam **R**econstruction from **S**equential irradiation **S**cenarios (***SIRS***) can only be assured when alpha and gold fluences are incremented by an infinitesimal amount so as not to bias the damage state towards the last irradiating ion of the sequence. To prove this hypothesis, a new multiple sequential ion irradiation experiment was performed in which alpha and gold fluences were incremented by 1 × 10^15^ He ions cm^−2^ and 2 × 10^12^ Au ions cm^−2^ respectively to reconstruct the 4 × 10^15^ He & 8 × 10^12^ Au ions cm^−2^ simultaneous irradiation scenario. The reconstruction scheme is shown below:

(i) **He + Au SIRS:** The irradiation sequence was started by alpha irradiation and finished by gold irradiation. Following irradiation sequence was used for the reconstruction:

1 × 10^15^ He + 2 × 10^12^ Au + 1 × 10^15^ He + 2 × 10^12^ Au + 1 × 10^15^ He + 2 × 10^12^ Au + 1 × 10^15^ He + 2 × 10^12^ Au.

(ii) **Au + He SIRS:** The irradiation sequence was started by gold ion irradiation and finished by alpha irradiation. Following irradiation sequence was used for the reconstruction:

2 × 10^12^ Au + 1 × 10^15^ He + 2 × 10^12^ Au + 1 × 10^15^ He + 2 × 10^12^ Au + 1 × 10^15^ He + 2 × 10^12^ Au + 1 × 10^15^ He.

where;


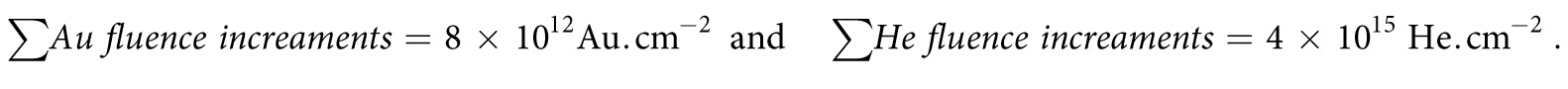


**Figure 10:**
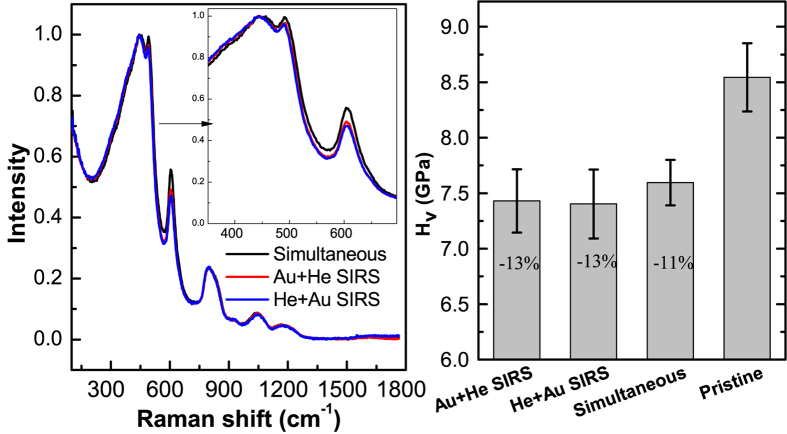
Comparison of simultaneous ion beam reconstruction (SIRS) with simultaneous ion beam irradiation. (**a**) Non-polarized Raman spectra of a-silica; (**b**) Vickers micro-hardness.

Both irradiation sequences i.e. Au + He SIRS and He + Au SIRS were studied so as to check if the last irradiating ion was introducing any bias in the results. This is important to confirm that the fluence increments are small enough and fulfil the infinitesimal fluence increment criterion. The Raman spectra and micro-hardness results of these reconstructions and that of the simultaneous irradiation scenario are shown in Fig. 10. The SIRS results show a very good agreement with each other as well as with the simultaneous irradiation scenario. This provides an unambiguous proof that the simultaneous ion beam can be reconstructed from multiple sequential irradiation scenarios. It also shows that no new phenomenon is present during simultaneous beam irradiation of a-silica and that there is no space-time overlap between the ions at the studied flux levels (see Table 1).

In summary, these results highlight many important points critical to understand the damage evolution during simultaneous particle irradiation. First of all, the study on amorphous silica shows that the simultaneous particle irradiation scenarios can be reconstructed from multiple sequential irradiation scenarios. Therefore, the study should be extended to other materials as well. The cumulative damage contains a contribution from single as well as double beam irradiated regions. There is no time-space correlation between different ion impacts and consequently no new phenomenon of particle-matter interaction or alteration of defect kinetics occurs during simultaneous ion beam irradiation of silica glass even at the very high fluxes. However, further studies with different ion pairs and other materials are needed to understand when time-space correlations occur. The importance of the simultaneous irradiation lies in the fact that in most of the multi-particle irradiation environments the materials experience slow damage buildup due to many particles rather than sequential damage buildup due to the particles. This slow incremental damage buildup due to many particles is what is best captured by the simultaneous particle irradiation. This understanding would offer a very viable and convenient route to simulate complex irradiation problems where materials are subjected to simultaneous irradiation with many particles for which the simultaneous irradiation facilities either do not exist or do not allow energy, flux and sample dimension flexibility. In such cases, the SIRS process could be conveniently applied. Immediate applications are simultaneous neutron and swift heavy irradiations experienced by fuel and cladding in fission reactors, nuclear waste matrices subjected to intense irradiation with electrons, gamma rays, alpha particles and recoil nuclei during few hundred years after disposal, spallation target vessels, primary beam dumps used in accelerators which are subjected to multiple particle irradiations over time, and space vehicles and meteorites which suffer high energy particle irradiations. Whether the SIRS can be generalized and extended to a wide range of materials remains to be tested. It is important to bear in mind that the fluxes experienced by many materials during operational conditions are orders of magnitude smaller than the ones employed in external irradiation facilities. Therefore, it makes it more likely to generalize the SIRS process. In principle, one can always choose very low fluxes so that no time-space correlation between different damage events exists.

The study also showed that the low electronic energy loss (~0.3 keV/nm) can cause partial repair of the pre-existing defects in amorphous silica and that the repair results from a low temperature non-melting thermal spike accompanying the electronic energy loss. Whether there is a lower and upper stopping power threshold where the defect recovery ceases to exist is not known at the moment. Therefore, more studies on sequential irradiation with lower/higher electronic energy loss ions are needed in near future. Since the recovery process was attributed to the formation of a non-melting thermal spike, it is therefore reasonable to hypothesize that below ion track threshold electronic energy loss should lead to a recovery process. It is important to highlight that the recovery effects become dominant and measurable only when the net recovery in the pre-damaged material exceeds the net damage formation in the pristine material. This makes it important to make detailed sequential ion irradiation studies as a function of the pre-damage level to fully understand the nature of the interaction mechanism. The ionization induced recovery also highlights the need to pursue studies on the impact of low electronic energy loss on the shape and size of pre-existing ion tracks in radioisotope bearing minerals used for ion track dating and thermogeochronology. Such studies could be important to understand the nature and extent of ion track annealing and its impact on the track morphology and counting.

## Methods

### Ion irradiations

Optically polished samples (3 × 3 × 0.5mm) of amorphous silica (a-silica) were irradiated with 2 MeV alpha particles and 14 MeV gold ions at JANNUS Saclay, France under mono and double beam irradiation scenario. During mono beam irradiation, samples were irradiated with either alpha particles (He) or gold ions (Au) to various fluences (see also section-3 in the SM). For sequential ion irradiation, samples pre-irradiated with a given alpha fluence were subsequently irradiated with gold ions (He + Au) and vice versa (Au + He). In addition, samples were also simultaneously irradiated with alpha particles and gold ions (He & Au). The general irradiation plan is schematically shown and the various fluences are tabulated in Table S1 in the Supplementary material. Other irradiation details are shown in Table 1. For the SIRS experiment, the samples were irradiated under same conditions as used for the rest of the irradiations. Following irradiation with one type of the ion, the beam was stopped and the second type of the ion beam was turned on. This process was repeated until the required fluences were attained.

### Raman spectroscopy

Horiba HR800 micro Raman spectrometer using 532 nm excitation laser was used for non-polarized confocal Raman spectroscopy. The depth, lateral and spectral resolution were 1.6 μm, 1 μm and 1.7 cm^−1^ respectively. The spectra were acquired at three different locations on the irradiated sample surface to check the damage homogeneity. For a given fluence, two samples were analyzed. The errors shown on the figures show a standard deviation of three independent measurements.

### Micro hardness testing

Vickers hardness was measured using Anton Paar MHT10 micro indenter fitted with a pyramidal diamond tip (half apex angle of 68°). Since the ion range was a few micrometers only, pristine and irradiated samples were indented with a low load of 0.196 N (20gf) so as to access the irradiated region only^8^. The indents were observed with Olympus BX51 optical microscope using 100x magnification objective. The value of the hardness was obtained from an average of 25 images (five indents and 5 images per indent).

## Additional Information

**How to cite this article**: Mir, A. H. *et al*. Understanding and simulating the material behavior during multi-particle irradiations. *Sci. Rep.* **6**, 30191; doi: 10.1038/srep30191 (2016).

## Supplementary Material

Supplementary Information

## Figures and Tables

**Table 1:** Irradiation conditions and stopping power data.

Ion	Range (μm)	Sn (keV.nm^−1^)	Se (keV.nm^−1^)	Se/Sn	Flux (ions.cm^−2^s^−1^)
2 MeV He^+^	6.7	3 × 10^−4^	0.3	10^3^	1 × 10^13^
14 MeV Au^6+^	3.4	1.2	2.5	2	2 × 10^10^

The gold and alpha Ion beams were incident at an angle of 15° to the sample normal. The vacuum in the chamber was maintained at 2 × 10^−7^ torr. The electronic (Se) and nuclear (Sn) stopping powers were calculated using SRIM2008 code (see section-5 in the SM for SRIM profiles and information about He bubble formation in SiO_2_).
