# Supplementary material for: Understanding and simulating the material behavior during multi-particle irradiations
Source: Sci Rep. 2016 Jul 28;6:30191. doi: 10.1038/srep30191 (PMC4964566; doi:10.1038/srep30191)
Supplement: Supplementary Information [file srep30191-s1.pdf]

Supplementary Information: Understanding and simulating the material behavior during multi-particle irradiation scenarios

A H Mir[1], M Toulemonde[2], C Jegou[1], S Miro[1], Y Serruys[3], S Bouffard[2], S Peuget[1]

1. Irradiation induced structural changes in amorphous silica (a-silica)

The irradiation of a-silica with either alpha particles or gold ions resulted in changes in the population of three (D2 peak at 602 cm^{-1}) and four member silica rings (D1 peak at 490 cm^{-1}). Apart from this, changes in other regions of the Raman spectra were also seen. The frequency of the R band (440 cm^{-1}) which gives an estimate of the average Si-O-Si bond angle increased. This suggests a decrease in the bond angle and a possible network densification[1]. More details about these kind of defects and the information one can obtain from such defect types can be found in[2,3]. It is interesting to highlight that the energy of formation of the three and four member rings is about 0.4±0.03 eV and 0.14±0.02 eV [2]. Owing to the low energy loss of the alpha particles and low energy of formation of four member rings, it is probably not surprising to see a preferential formation of four member rings during alpha particle irradiation.

The peaks at 1064 cm^{-1} (in phase motion of the oxygen atoms towards the Si atom/symmetric stretch of oxygen in Q^4 units; where Q^4 is a silica tetrahedron connected to four oxygen atoms) and 1190 cm^{-1} (motion of two oxygen atoms towards the Si atom and motion of other two oxygen atoms away from the Si atom involving also motion of the Si atom/asymmetric stretch of oxygen atoms in a Q^4 unit)[4,5] showed a downward frequency shift and broadening. These changes are consistent with increase in the network disorder.

Apart from the changes in these bands, two new peaks emerged at 933 cm^{-1} (origin uncertain) and 1552 cm^{-1} (molecular oxygen)[6] during gold ion irradiation. The origin of the peak at 933 cm^{-1} is not very well documented in the literature. It has been observed in a-silica samples with 1 to 3 percent of either B_2O_3 or TiO_2 and attributed to the vibrations of non-bridging oxygen atoms[17] (the band is polarized). Vibrational analysis of Si_2O_6 chains (each Si atom containing two non-bridging oxygen atoms-Q^2 unit) by Furukawa[18] has shown the possibility of a Raman and IR active peak at 932 cm^{-1}. Therefore, this band may be attributed to the symmetric stretching of Si atom with two bridging and two non-bridging oxygen atoms. Breaking the rings into chains or smaller fragments consisting of Q^2 units would require atomic displacements to disintegrate the rings either completely into linear chains and fragments or into smaller chains with attached Q^2 units. Alpha particle due to its low energy loss is probably not able to create such high extent of damage and consequently such structures were not observed during alpha irradiation.

The structural changes or the changes in ring statistics due to external stresses (radiation, pressure) are accompanied by the formation of a number of point defects. The low level of ionization due to photons and light ions can lead to direct ionization or excitation of electrons to anti-bonding orbitals whereas high electronic energy loss and ballistic collisions

[1] CEA, DEN, DTCD, SECM, LMPA BP 17171, 30207 Bagnols-sur-Ceze Cedex, France.
[2] CIMAP-GANIL (CEA-CNRS-ENSICAEN-Univ. Caen), BP 5133, 14070 Caen Cedex 5, France.
[3] CEA, DEN, Service de Recherches de Métallurgie Physique, Laboratoire JANNUS, F-91191 Gif-sur-Yvette, France.
Corresponding author : A.H. Mir (mirinamulhaq@gmail.com); S. Peuget (sylvain.peuget@cea.fr).

can lead to atomic displacements. These effects lead to the formation of defects like E’ centers (silicon atom connected to three bridging oxygen atoms and containing an unpaired electron in an sp3-like orbital/paramagnetic), NBOHC (non- bridging oxygen hole center. It is a silicon atom connected to three bridging oxygen atoms and one non-bridging oxygen atom which contains an unpaired electron/paramagnetic) and neutral oxygen deficient centers (like ODC) [8–10]. Therefore, both alpha as well as gold ion irradiation are expected to result in such defect types. A number of studies have been performed on such defects in pristine and ion irradiated a-silica for which readers are referred to[11–16] and the references therein. Thermal treatment of irradiated a-silica usually leads to a decrease of oxygen deficient centers (E’, ODC) and an increase in the population of oxygen rich defects like NBOHC and peroxy-bridges. The inter-conversion between different defect types usually depends on the concentration of each defect type and the temperature.

The mechanism of irradiation induced defect recovery; which was observed during Au+He sequential ion irradiation, is not clear. A detailed analysis of defects using optical absorption/photoluminescence/EPR is therefore needed to calculate the concentration of different defect centers following alpha irradiation of the gold pre-damaged a-silica. Nonetheless, some possible hypothetical mechanisms are listed below in Fig S. Each one of the mechanism must be checked for the energetics for which detailed ab-initio calculations are needed in future.

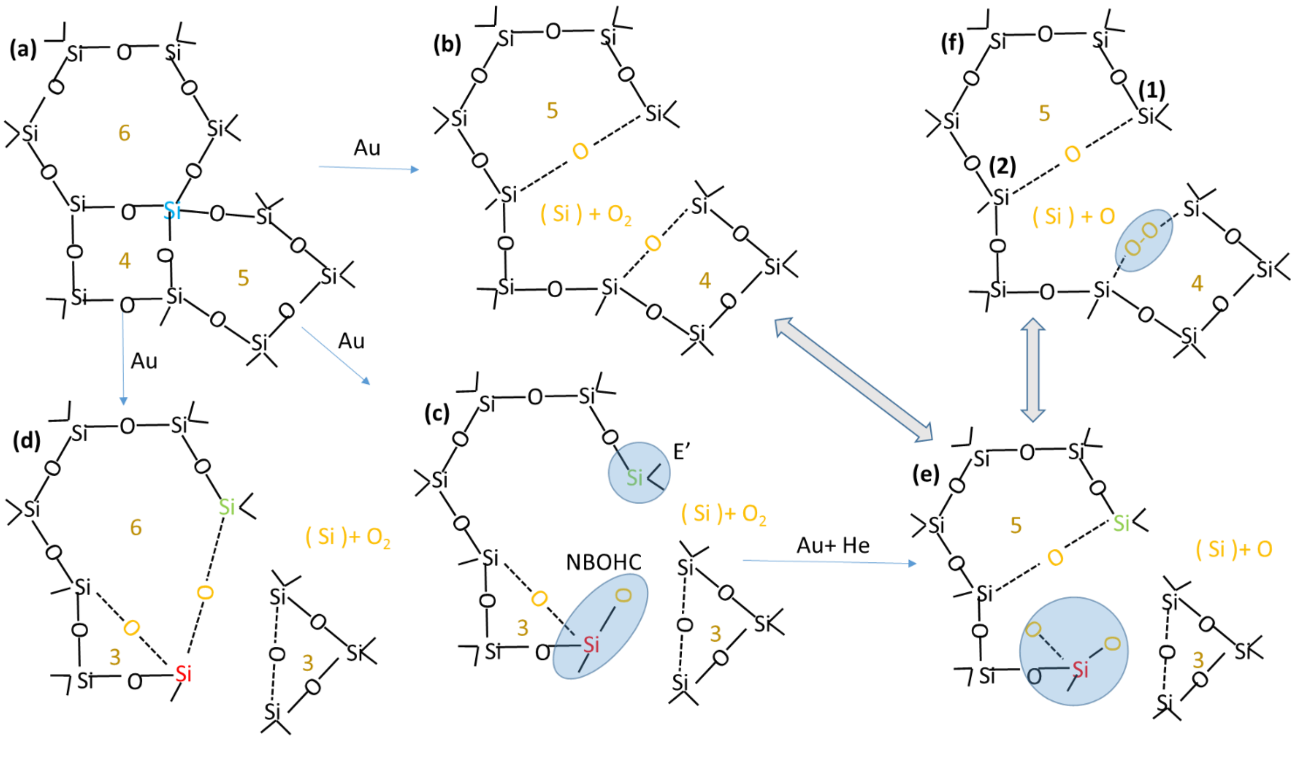

Figure-S: Hypothetical damage and recovery mechanisms after gold and alpha irradiation.

A hypothetical case of interconnected 6-4-5 member rings is shown in (a). A silicon atom knock out may lead to the formation of various final damaged states. Due to the randomness of the structure and damage events various defect structures may be formed during different damage events. Fig. (b) shows a transformation of the 6 and 5-member rings to 5 and 4-member rings respectively (the transition occurs through the formation of a penta-coordinated silicon). The four member ring is destroyed and interstitial silicon and molecular oxygen is formed. Figure (c) shows a possibility of

the formation of two 3-member rings and opening of the 6-member ring due to silicon displacement. Non-bridging oxygen hole (NBOHC/Q^3 unit) and E' defect centers are also formed. Note that the E'-NBOHC pair is unstable when in close proximity and charge transfer from E' to NBOHC results in self-annihilation of these defects and ring closure[17]. Therefore, for them to exist, they should be well separated. In ab-initio studies of photoionization of three member silica rings[17] the defect pair was found to be stable at a distance of about 6.3 A°. In figure (d), a deformed 6-member ring and two 3-member rings are formed. As E' and NBOHC are usually found in irradiated a-silica, therefore, the effect of gold followed by alpha irradiation (Au+He) is shown on the defect state shown in figure (c). In going from (c) to (e), an atomic oxygen results due to alpha induced ionization of molecular oxygen. Ring closure leads to the formation of a five member ring. In addition, a Q^2 unit (Silicon bonded to two bridging oxygen atoms and two non-bridging oxygen atoms) is also formed. It was proposed that the Raman peak at 933 cm^{-1} is possibly a Q^2 unit and was observed to be destroyed by alpha irradiation. Therefore, state (c) is very unlikely to be favoured upon alpha irradiation. The transformation of state (e) to state (b) is possible, but there is no overall change in the consumption of the oxygen atoms. Therefore, state (f) is most likely possible than states (e) and (b) as it is consistent with the formation of higher order rings upon alpha irradiation and annihilation of the molecular oxygen. Note that a peroxy-bridge is formed in state (f) which leads to the ring closure. If however, one starts from a defect type like ODC (Si-Si) in state (c), then incorporation of an oxygen atom resulting in the formation of a Si-O-Si link can lead to ring closure as well as consumption of oxygen atoms. At this point it is important to highlight that as long as the silicon atoms stay as an interstitial, the excess oxygen would exist in the form of oxygen rich defects, peroxy-bridges or molecular oxygen (and ozone). Since Raman spectroscopy showed that the molecular oxygen is destroyed upon alpha irradiation, therefore existing as oxygen rich centers and peroxy-bridges is the most likely case. The extent to which molecular oxygen is consumed should therefore depend on the concentration of the peroxy bridges (thermal treatments usually lead to the formation of peroxy bridges). An additional peroxy-bridge between Si (1) and Si (2) in figure (f) can consume one more oxygen atom. An increase in the concentration of oxygen rich defects and a decrease in the concentration of oxygen deficient centers has been observed in a-silica infused with oxygen at high pressure and then subjected to gamma irradiation (see chapter 6 in the thesis[18]) which is consistent with what is proposed above. The magnitude of the decrease in that study was found to be proportional to the gamma dose (^{60}Co) but the decrease became significant only after 5MGy. The minimum dose studied in our work is about 400 MGy corresponding to $2x10^{15}$ He ions cm^{-2}.

2. **Damage models**

Each ion that hits the sample creates certain level of damage around its path. In certain situations a single impact may create maximum possible damage level such that any additional impacts at the same location do not create any further damage. The impact zone around the ion path that is transformed by the ion therefore represents the damage cross section of the ion (σ). The defect generation in such a scenario can be fitted with a single impact damage model. In certain circumstances a single impact may not be enough to transform the material to its final damage state and multiple impacts may be need. In this case a multiple impact damage model may be needed. During a multiple impact damage process, a material may be transformed from initial state to a first damage state 1 with a certain cross section

(σ1) and it may then be transformed from state 1 to the final damage state (or state 2) with a different damage cross section (σ2). It is also possible that the damage cross section does not change. The kind of damage impact model that one should use depends on the trends in the damage evolution as a function of the ion fluence and it is not possible to determine a priori. More information about damage models can be found in [19,20]

A general multi-impact damage model with a constant damage cross section can be written as:

$$D_f = 1 - \left(\sum_{k=0}^{n} \frac{(\sigma\emptyset)^k}{k!}\right).exp^{(-\sigma\emptyset)}$$

Where k=0, 1, 2…….. represent single, double, triple……..damage impact model. It can also be written as

$$D_f = 1 - \left(1 + \sigma\emptyset + \frac{(\sigma\emptyset)^2}{2!} + \frac{(\sigma\emptyset)^3}{3!} + \cdots + \frac{(\sigma\emptyset)^n}{n!}\right).exp^{(-\sigma\emptyset)} \qquad (1)$$

For a single impact, the equation reduces to

$$D_f = 1 - exp^{(-\sigma\emptyset)} \qquad (2)$$

The quantity D_f called as the damage fraction represents the ratio of change in a given parameter at a given fluence (peak area, hardness etc.) to the maximum observed change in that parameter/saturation value.

If one assumes that the Au ion impact results in a damage zone with an average damage cross section σ, then the damage evolution can be described using equation (1).

Applying the same to the D_2 peak area evolution, one can write $D(\emptyset)=D_0+\Delta D_{max}\,[1-exp^{(-\sigma\emptyset)}]$, where D_0 is the D_2 peak area of the unirradiated sample, D (Ø) is the D_2 peak area at a fluence Φ and ΔD_{max} is the maximum D_2 peak area change and $D_f=\Delta D(\Phi)/\Delta D_{max}$ is the damage fraction.

D_f=0 for unirradiated samples and D_f=1 for completely damaged sample (saturation damage state). The same damage impact model can be applied to the hardness variation where D in above equation represents the hardness.

As described in the manuscript, gold and alpha particles caused an increase of the D2 peak area by 18 and 9 units respectively. Similarly, gold and alpha irradiation caused a decrease of the hardness by 21% and 12% respectively. In general, the alpha induced changes are only about half as that of the gold ion irradiation induced changes. Therefore, on a scale of 0 to 1, the maximum alpha damage level is close to 0.5 and gold damage level is 1. The fits of single impact damage model to the variation of D2 peak are shown in inset-I in Fig. 1 and Fig. 2 for gold and alpha irradiation respectively in the main article. Note that the saturation level of the alpha particle is shown as 0.5 which is relative to the saturation level of the gold ion irradiation.

A comparison of single and double impact damage models is shown in Fig. S0 for the case of gold and alpha irradiation. It clearly shows that there is no strong motivation to use a double impact model. In general, very precise

data at low fluences is needed to decide the damage model due to slow damage growth at early stage of a double impact process.

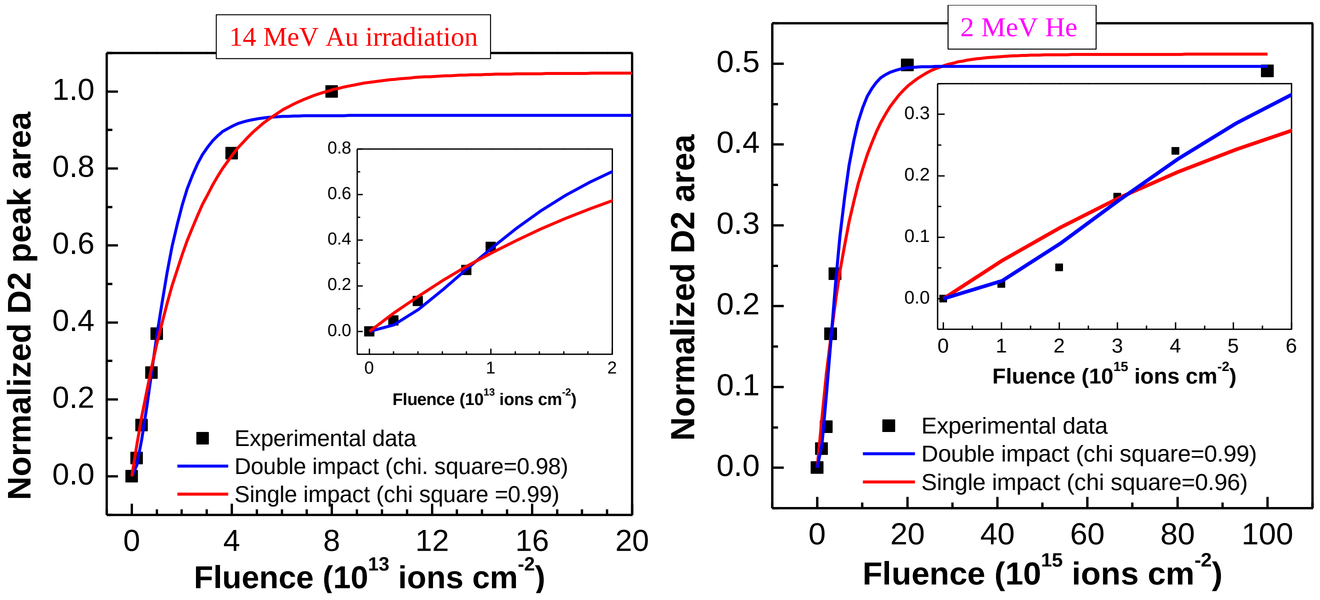

Figure-S0: Comparison of single and double impact damage models.

3. Irradiation Details

3x3x0.5 mm^3 samples of amorphous silica (a-silica) were irradiated with 14 MeV gold ions and 2 MeV alpha particles at room temperature. The samples where irradiated under various irradiation scenarios viz: i) Irradiation with either gold ions (Au) or alpha particles (He) to understand the response of the pristine a-silica to these ions; ii) sequential irradiation with alpha particles and gold ions to understand the response of the pre-damaged a-silica. During alpha followed by gold ion irradiation (He+Au) samples were first irradiated with various alpha fluences. Then the samples with a given alpha fluence were irradiated with various gold ion fluences. This allowed studying the response of the alpha pre-irradiated a-silica as a function of the pre-damage level. Same study was performed for gold followed by alpha irradiation sequence (Au+He); and iii) simultaneous irradiation with alpha and gold ions. The various irradiation scenarios and different fluence combinations are shown in Table-S1.

Table-S1: Various irradiation scenarios and studied fluence combinations.

Irradiation scenario	Ion fluence (ions.cm^{-2})
Alpha **(He)**	2e15, 4e15, 2e16, 1e17 and 1e15, 3e15 on which only Raman spectroscopy was performed. These two data points can be seen in Fig. 4 and Fig. 5 in the article).
Gold **(Au)**	2e12, 4e12, 8e12, 2e13, 4e13, 8e13
He followed by Au irradiation sequence. **(He+Au)**	2e15He+4e12Au, 2e15He+8e12Au, 2e15He+1e13Au+2e15He+4e13Au
	4e15He+4e12Au, 4e15He+8e12Au, 4e15He+1e13Au+4e15He+4e13Au
	2e16He+4e12Au, 2e16He+8e12Au, 2e16He+1e13Au+2e16He+4e13Au
Au followed by He irradiation	4e12Au+2e15He, 4e12Au+4e15He, 4e12Au+2e16He, 4e12Au+1e17He

sequence. **(Au+He)**	8e12Au+2e15He, 8e12Au+4e15He, 8e12Au+2e16He, 8e12Au+1e17He
	1e13Au+2e15He, 1e13Au+4e15He, 1e13Au+2e16He, 1e13Au+1e17He
	4e13Au+2e15He, 4e13Au+4e15He, 4e13Au+2e16He, 4e13Au+1e17He
Simultaneous irradiation with He and Au. **(He & Au)**	2e15He & 4e12Au,<br>4e15He &8e12Au,<br>4e13Au & 2e16He.

This is shown graphically below:

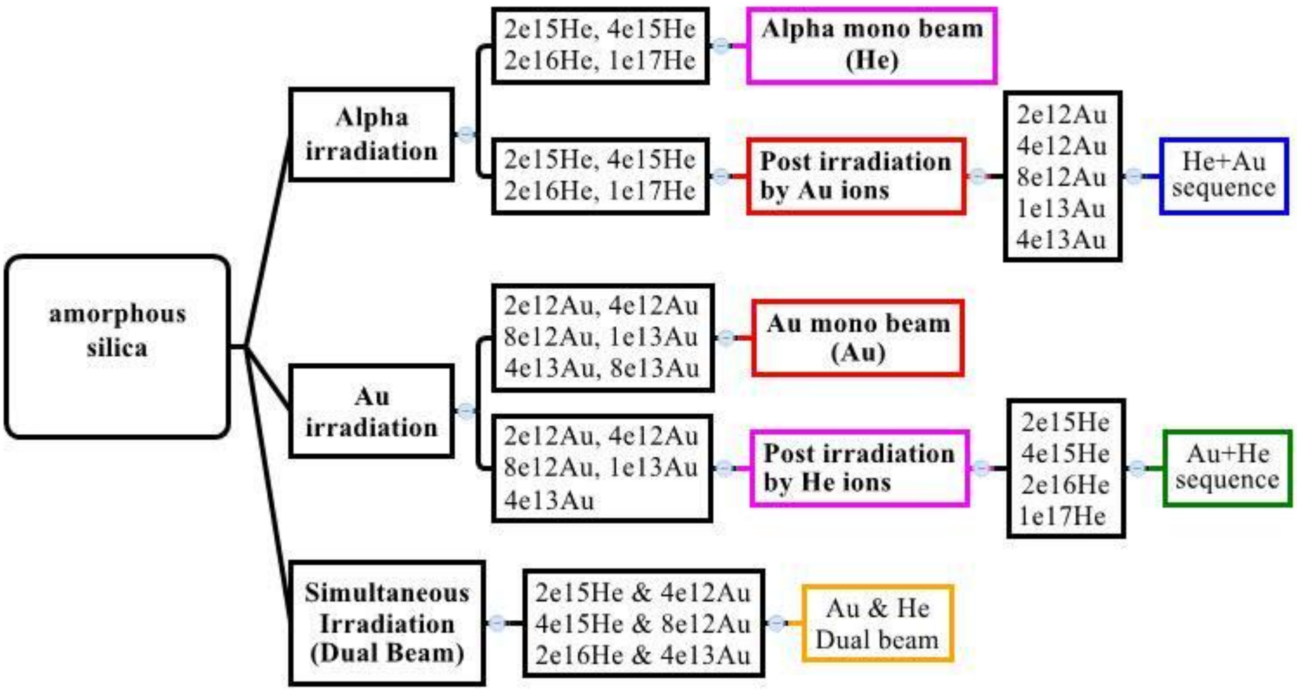

Description: Alpha irradiation (pink), Au irradiation (red), alpha followed by the Au irradiation (blue), Au followed by the alpha irradiation (green) and simultaneous irradiation with alpha and gold ion beams (orange). Various samples with a given alpha pre-irradiation fluence were irradiated with different gold fluences as shown above. Similarly gold pre-irradiated samples were irradiated with various alpha fluences. For details see Table-S1 in the supplementary material.

4. Supplementary Figures

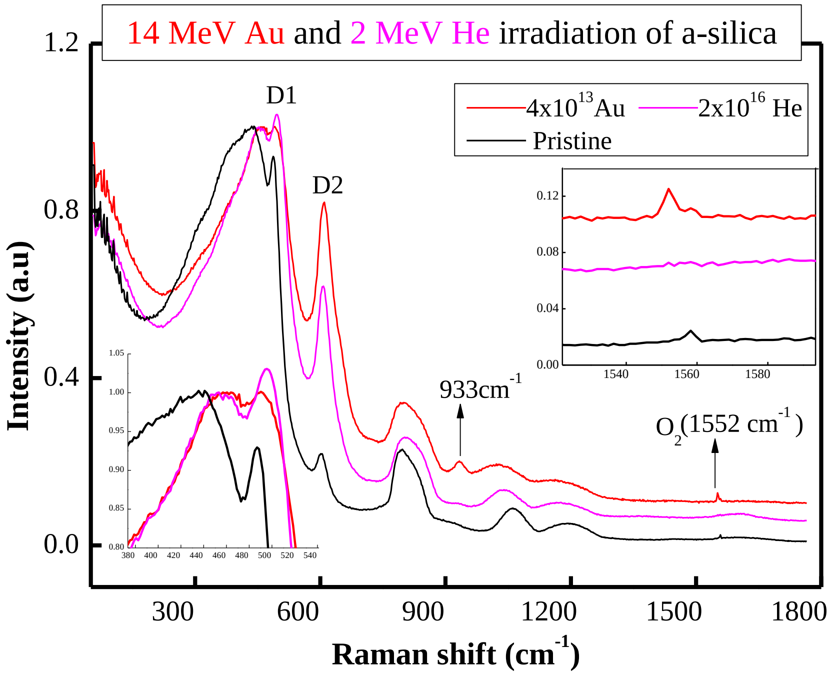

Figure S1: Non-polarized Raman spectra of a-silica: Comparison of 2 MeV alpha (pink) and 14 MeV gold ion (red) irradiations. The Raman spectra are shown for the saturation damage states. The raw spectra can be downloaded at https://drive.google.com/open?id=0B-sQarnPvp5taFc3Y0RraUJJYnM

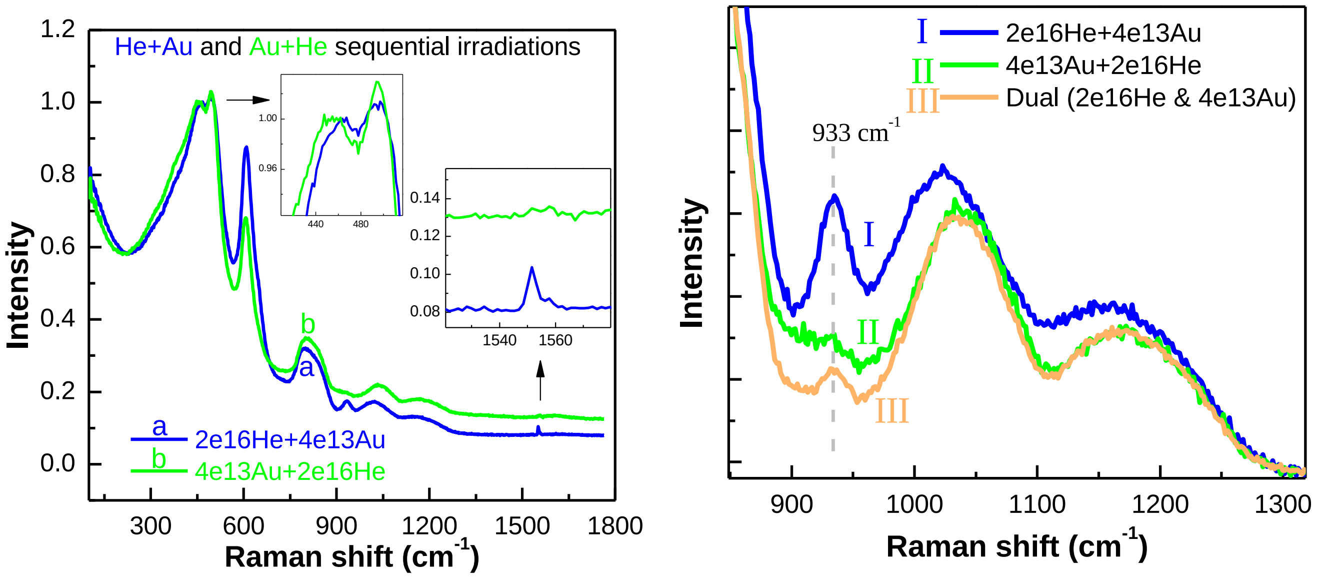

Figure S2: Non-polarized Raman spectra of irradiated a-silica. (Left) Comparison of He+Au (Blue-a) and Au+He (Green-b) sequential irradiation scenarios. (Right) Comparison of He+Au (Blue-I), Au+He (Green-II) and dual (Orange-III) irradiation scenarios in the region from 850 cm^{-1} to 1300 cm^{-1}.

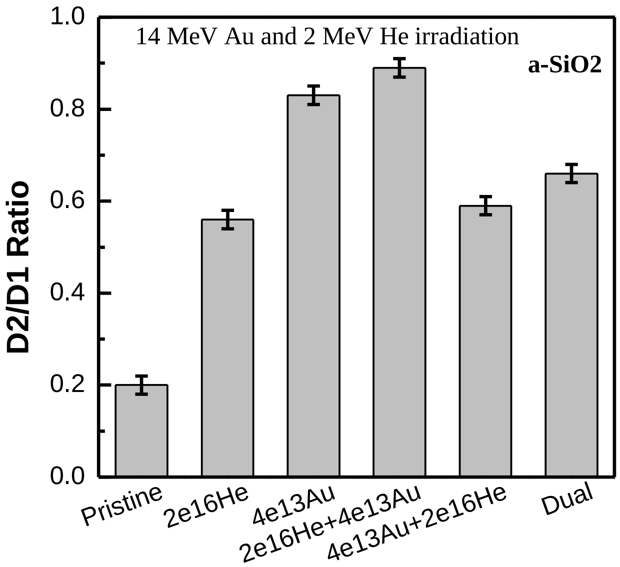

Figure S3: D2/D1 peak intensity ratio. Comparison of sequential and simultaneous ion irradiation scenarios for the saturation damage state.

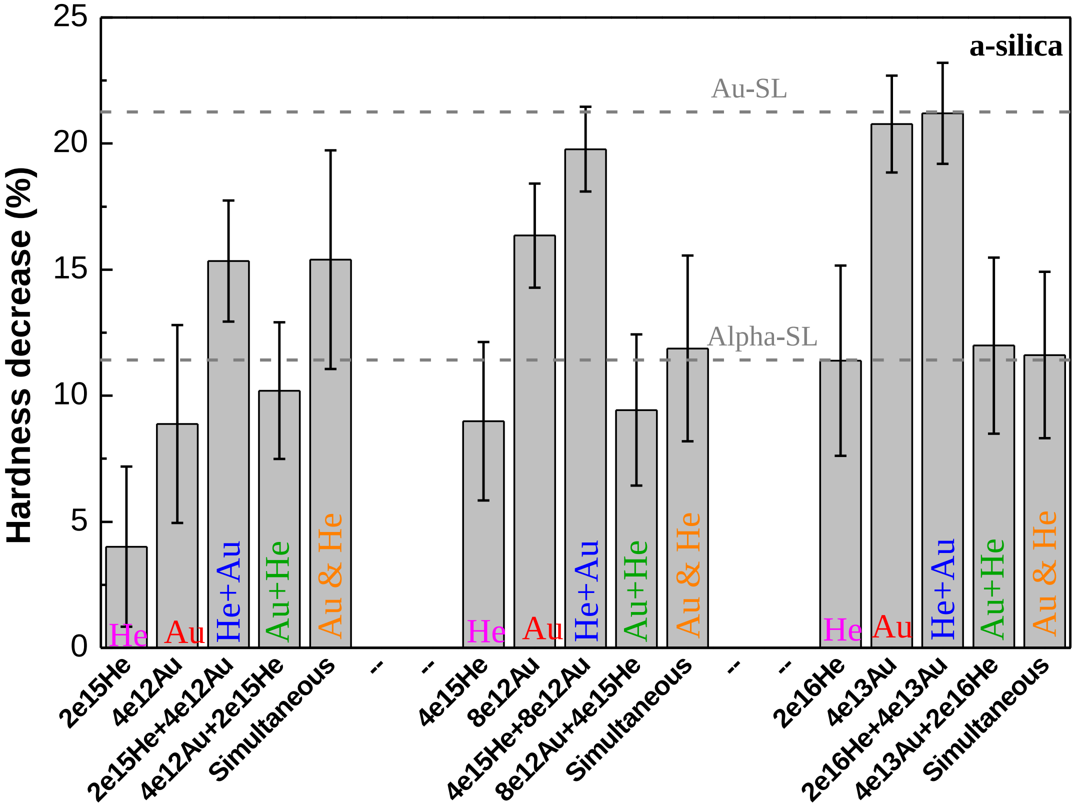

Figure S4: Comparison of various irradiation scenarios at different damage levels. The error bars show a standard deviation of 25 measurements.

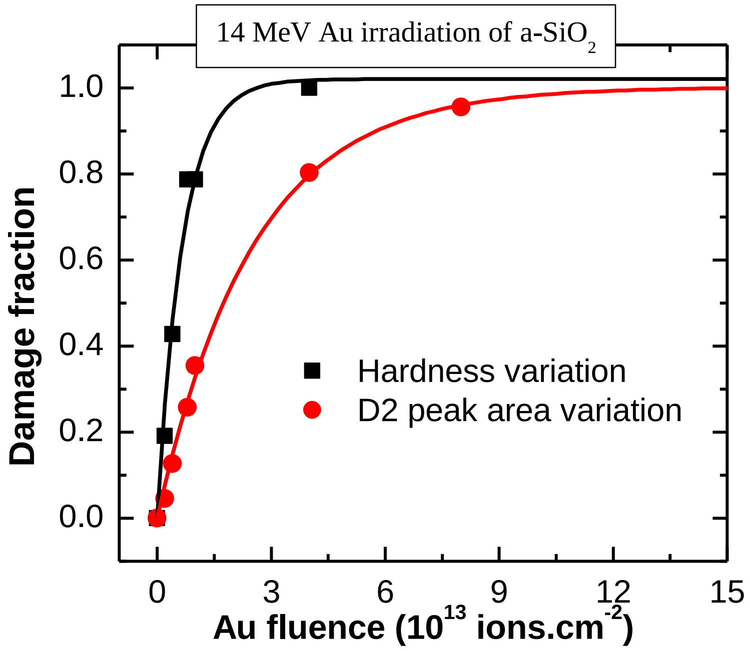

Figure S5: Comparison of the variation of micro hardness and D2 peak area (three member silica rings) as a function of gold ion irradiation fluence. The data is normalized to 1 for the purpose of comparison and is fitted with single impact damage model.

Thermal spike calculations for 14 MeV gold ion irradiation.

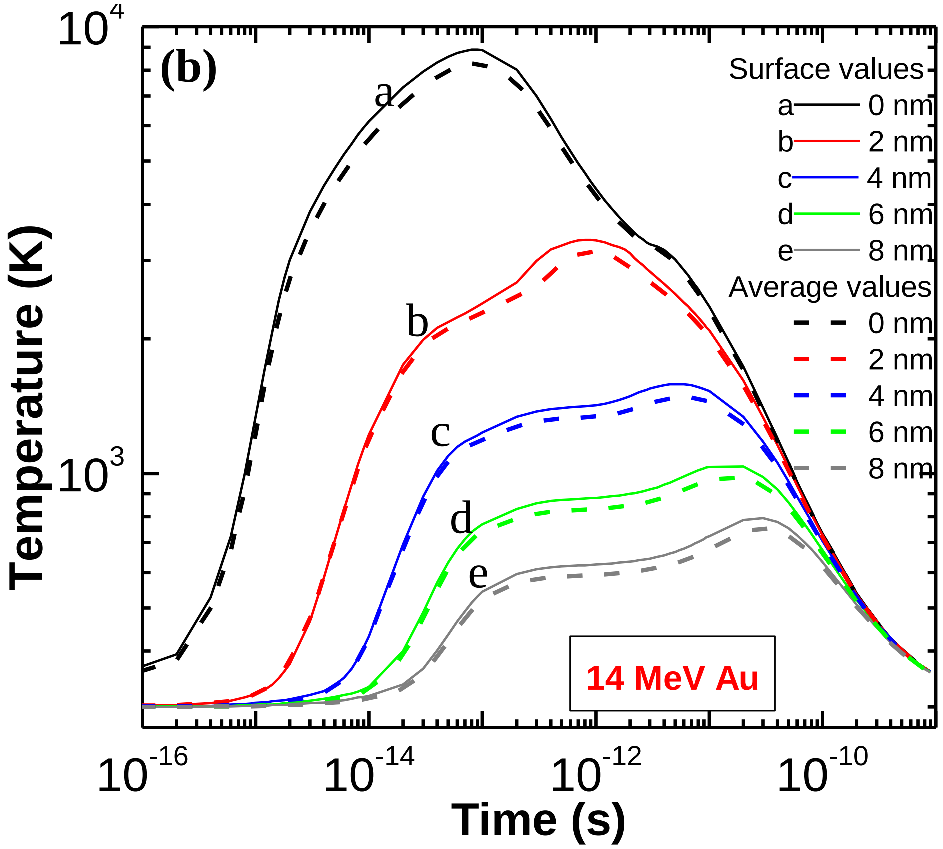

Figure S6: Inelastic thermal spike calculations of 14 MeV gold in amorphous silica. The solid lines (surface values) show the temperature profiles for surface stopping power and dashed lines (average values) show the temperatures profiles for average stopping power in 1.6 µm depth which corresponds to the Raman depth resolution. The calculations were performed using an electron-phonon coupling constant of 3 nm. For more details, readers are referred to[21].

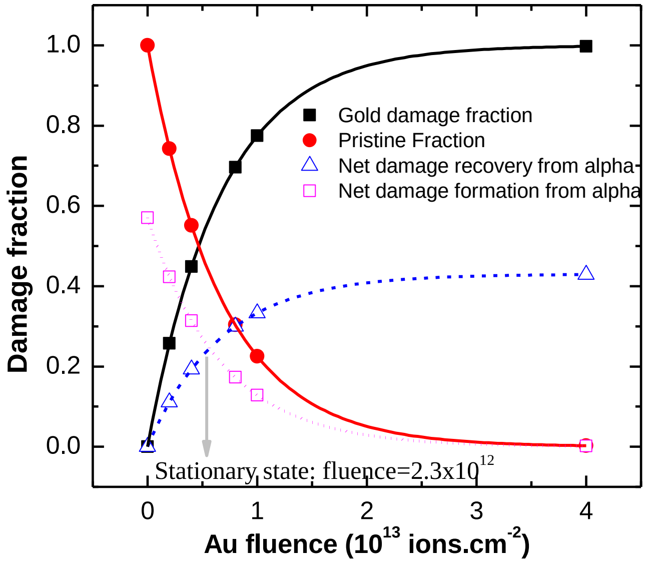

Figure S7: Probability of alpha particles to encounter gold pre-damaged zone (black) and pristine zone (red). The alpha induced recovery is proportional to the black curve and alpha fluence whereas alpha induced damage formation is proportional to the red curve and alpha fluence. On a scale of 0 to 1 (1 being the damage fraction of gold ions) the blue dashed line shows the overall extent of recovery caused by alpha irradiation of the gold pre-damaged zone and pink dashed line shows the overall damage formation by the alpha irradiation of the pristine zone. The point of intersection of these two lines defines whether damage formation or damage recovery would be apparent.

Description: The black line shows the gold damage fraction. The probability of alpha induced recovery is also proportional to D_f. the probability of damage formation due to alpha irradiation of the pristine zone is proportional to 1-D_f as shown with black line. The values of "d" and "r" are 0.57 and 0.43 (from Fig. 7 in the manuscript). The dashed lines in pink and blue are $(1-D_f)*d$ and D_f*r. Some cases are discussed below

i). $D_f=0$: alpha irradiation only results in damage formation in the pristine part and there is no recovery. Therefore the damage level is 0.57 and recovery level is 0 as shown by pink and blue lines respectively.

ii). $D_f=1$: alpha irradiation causes maximum possible recovery in the gold pre-damaged zone and no addition damage formation. This is shown by blue and pink lines for a fluence of $4x10^{13}$ $Au.cm^{-2}$ (saturation damage state).

iii). $0<D_f<1$: for any intermediate damage fraction, there is a competition between damage formation in the pristine zone and damage recovery in the pre-damaged zone. A stationary state is reached for a gold fluence of $2.3x10^{12}$ ions.cm^{-2} (D_f=0.57).

It is important to highlight that when the recovery level in a given material is small, it is difficult to know if the damage states depend on the irradiation sequence of the ions. Detailed studies on SiC have shown that the extent of ionization induced recovery is proportional to the electronic stopping power of the ions (see Fig. 2 in Y. Zhang et al [22]) . Therefore, it is important to perform detailed sequential ion irradiation studies to ascertain if the recovery mechanisms are present or not and what are the necessary conditions for the damage recovery to occur.

5. Ion distribution and discussion on possibility of He bubble formation.

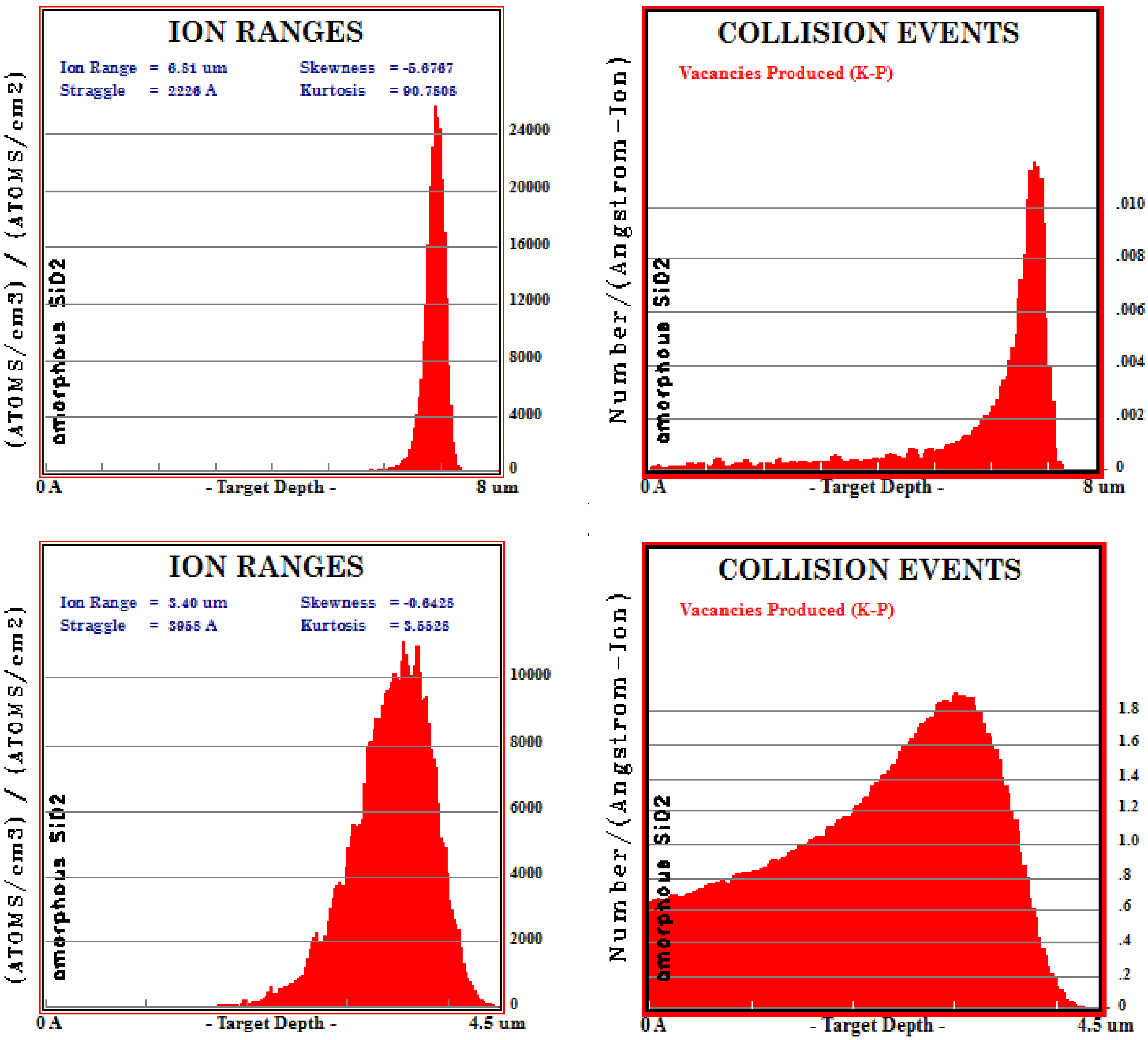

Fig.S8: (top panel) the range and collision events of 2 MeV He in amorphous silica; (bottom panel) range and collision events of 14 MeV gold in amorphous silica.

Absence of He bubbles in SiO_2 at the studied fluence: The concentration of various Helium solubility sites in amorphous SiO_2 is about $2.3x10^{21}cm^{-3}$ (*the atomic density is about $6.6x10^{22}$*)[23]. The solubility of Helium in SiO_2 at room temperature and pressure (NPT) is about 0.025[24] which translates into $6.8x10^{17}$ cm^{-3} He atoms in the SiO_2 matrix[1] (*the number of occupied sites in relation to the number of available sites at NPT is therefore only about $3x10^{-4}$*)[23].

Now the steady state atomic concentration (C) in a sample during implantation is given by[25]

$$C = \frac{ØR(T - R)}{DTN}$$

Where, Ø is the flux (*10^{13} $He.cm^{-2}.s^{-1}$ in our case*), R is the range of the He ions (*6.7 μm*), T is the sample thickness (*1 mm*), D is the He diffusion coefficient (*~10^{-7} $cm^2.s^{-1}$ at room temperature*)[25,26] and N is the atomic density of the sample (*$6.6x10^{22}$*). Considering the values of these parameters during our experiment, the steady state He atomic concentration is about 10^{-8} (*~$6.6x10^{14}$ He atoms)*. This shows that the steady state concentration is far less than the He solubility limit in SiO_2 matrix. Consequently no He bubbles can be expected to be present. In general, the He bubbles start forming when all the available sites are occupied allowing the additional He atoms to cause over saturation.

As a matter of fact, a number of experiments have been performed in this direction by various researchers and owing to the large He diffusion coefficient, no helium bubbles have been observed even after irradiation with $4x10^{17}$ $He.cm^{-2}$[26–28]. Furthermore, the diffusion coefficient is not affected by the irradiation damage[25] and no microstructural changes[28] have been observed even at fluences an order of magnitude higher than in our study. In addition, studies on other ion and self-irradiated complex glasses have also shown that the glass microstructure at such fluence levels does not change due to radiation damage[29].

Therefore, from a theoretical as well as experimental perspective, we do not expect to have any He bubbles in our samples and we rule out any effects that might arise due to the bubble formation.

References

1. Furukawa, T. Raman spectroscopic investigation of the structure of silicate glasses. III. Raman intensities and structural units in sodium silicate glasses. *J. Chem. Phys.* **75,** 3226 (1981).

2. Awazu, K. & Kawazoe, H. Strained Si–O–Si bonds in amorphous SiO[sub 2] materials: A family member of active centers in radio, photo, and chemical responses. *J. Appl. Phys.* **94,** 6243 (2003).

3. Galeener, F. & Jr, J. M. Vibrational dynamics in 18-O substituted vitreous SiO2. *Phys. Rev. B* **23,** 5527–5530 (1981).

4. Spiekermann, G., Steele-MacInnis, M., Schmidt, C. & Jahn, S. Vibrational mode frequencies of silica species in SiO2-H2O liquids and glasses from ab initio molecular dynamics. *J. Chem. Phys.* **136,** 154501 (2012).

5. Sarnthein, J., Pasquarello, A. & Car, R. Structural and Electronic Properties of Liquid and Amorphous SiOz. An Ab Initio Molecular Dynamics Study. *Phys. Rev. Lett.* **74,** 4682–4685 (1995).

6. George, F. & Lederman, S. Species concentration measurements utilizing Raman scattering of a laser beam. *AIAA J.* **9,** 309 (1969).

7. Kato, D. Raman spectrometric determination of additive concentration in high-silica-content glasses. *J. Appl. Phys.* **47,** 2050 (1976).

8. Griscom, D. L. The electronic structure of SiO2: A review of recent spectroscopic and theoretical advances. *J. Non-Crystalline Solids* **24,** 155–234 (1977).

9. Devine, R. A. B. & Arndt, J. Correlated defect creation and dose-dependent radiation sensitivity in amorphous Sio2. *Phys. Rev. B* **39,** 5132–5138 (1989).

10. Skuja, L. Optically active oxygen-deficiency-related centers in amorphous silicon dioxide. *J. Non. Cryst. Solids* **239,** 16–48 (1998).

11. Ma, M. *et al.* Color center formation in silica glass induced by high energy Fe and Xe ions. *Nucl. Inst. Methods Phys. Res. B* **268,** 67–72 (2010).

12. Nagata, S. *et al.* Luminescence characteristics and defect formation in silica glasses under H and He ion irradiation. *J. Nucl. Mater.* **367-370,** 1009–1013 (2007).

13. Yang, T. *et al.* The transformation balance between two types of structural defects in silica glass in ion-irradiation processes. *J. Non. Cryst. Solids* **357,** 3245–3250 (2011).

14. Kajihara, K., Hirano, M., Skuja, L. & Hosono, H. Intrinsic defect formation in amorphous SiO2 by electronic excitation: Bond dissociation versus Frenkel mechanisms. *Phys. Rev. B* **78,** 094201 (2008).

15. Birtch, E. M. & Shelby, J. E. Annealing of hydrogen-impregnated and irradiated vitreous silica. *J. Non. Cryst. Solids* **349,** 156–161 (2004).

16. Arai, K. *et al.* Evidence for pair generation of an E' center and a nonbridging oxygen-hole center in y-ray-irradiated iluorine-doped low-OH synthetic silica glasses. *Phys. Rev. B* **45,** 10818 (1992).

17. Bernasconi, D. D. M. Ab-initio simulation of photoinduced transformation of small rings in amorphous silica. 5–8 (2004).

18. Nuccio, L. Diffusion of small molecules in amorphous SiO2 : effects on the properties of the material and on its point defects. (2009).

19. Gibbons, J. F. Ion Implantation in Semiconductors-Part II : Damage Production and Annealing. in *Proc. IEEE* **6,** 1062–1096 (1972).

20. Weber, W. J. Models and mechanisms of irradiation-induced amorphization in ceramics. *Nucl. Inst. Methods Phys. Res. B* **166-167,** 98–106 (2000).

21. Toulemonde, M. *et al.* Synergy of nuclear and electronic energy losses in ion-irradiation processes: The case of vitreous silicon dioxide. *Phys. Rev. B* **83,** 054106 (2011).

22. Zhang, Y. *et al.* Ionization-induced annealing of pre-existing defects in silicon carbide. *Nat. Commun.* **6,** 8049 (2015).

23. Kuhtetskiy, S. V., Fomenko, E. V. & Anshits, A. G. Molecular dynamics simulation of helium diffusion in vitreous silica. *J. Non. Cryst. Solids* **443,** 47–53 (2016).

24. Doremus, R. H. Physical and Chemical Solubility of Gases in Fused Silica. *J. Am. Ceram. Soc.* **49,** 462–467 (1966).

25. Jung, P. Diffusion of implanted helium in Si and SiO2. *Nucl. Inst. Methods Phys. Res. B* **91,** 362–365 (1994).

26. Szakacs, G., Szilagyi, E., Paszti, F. & Kotai, E. Determination of migration of ion-implanted helium in silica by proton backscattering spectrometry. *Nucl. Inst. Methods Phys. Res. B* **266,** 1382–1385 (2008).

27. Krickl, R., Nasdala, L., Götze, J., Grambole, D. & Wirth, R. Alpha-irradiation effects in SiO2. 517–522 (2008).

28. Donnelly, S. E., Hinks, J. A., Pawley, C. J., Abrams, K. J. & van den Berg, J. A. An in-situ TEM study of the effects of 6 keV He ion irradiation on Si and SiO2. *J. Phys. Conf. Ser.* **371,** 1–4 (2012).

29. Peuget, S., Delaye, J.-M. & Jégou, C. Specific outcomes of the research on the radiation stability of the French nuclear glass towards alpha decay accumulation. *J. Nucl. Mater.* **444,** 76–91 (2014).
